# Attitudes towards death among health care professionals and their perceived well-being at Aga Khan University (AKU)

**DOI:** 10.1192/j.eurpsy.2023.2033

**Published:** 2023-07-19

**Authors:** N. Khalid, A. Chachar, S. Siddiqui, S. Khan

**Affiliations:** 1 Internal medicine, The Indus health network; 2 Psyciatry, Aga Khan univeristy hospital, Karachi, Pakistan

## Abstract

**Introduction:**

Death is a complex construct to understand as it is influenced by the perceptions that HCP may have regarding end of life. Understanding these perceptions helps in addressing death anxiety in HCP which can otherwise negatively influence physician well-being and patient interactions such as breaking bad news.

**Objectives:**

To identify association between attitudes towards death among HCP and their perceived well-being.

**Methods:**

This is a cross-sectional study on 109 HCP including nurses (n=29), physicians (n=43), resident (n=25) and interns (n=12) across various specialties at AKU. Death anxiety was assessed through the ***death attitude profile revised scale*** and its correlation was seen with the perception of one’s own wellbeing through ***Perceived well-being scale***. A semi-structured pro-forma was used to collect demographic data.

**Results:**

The results showed that ***death anxiety was highest in interns*** (150.83 ± 17.94) followed by nurses (139 ± 20.67), residents (137.84 ± 15.79) and physicians (137.99 ± 21.59) and ***perceived well-being was lowest in interns*** (71.00 ± 10.10) followed by nurses (72.41 ± 10.43), residents (74.16 ± 12.83) and physicians (75.98 ± 12.19). The results of this study demonstrated a negative correlation between death anxiety and perceived well-being.

**Conclusions:**

The negative correlation between death anxiety and perceived well-being suggest that health care professionals are most vulnerable in the preliminary years of their career. It is therefore recommended that psychology of death and dying is given equal weightage in medical curriculum to enable physicians deal effectively with the trauma of bereavement and loss relating to or patients.

**Disclosure of Interest:**

None Declared

